# Effects on nutritional care practice after implementation of a flow chart‐based nutrition support protocol in an intensive care unit

**DOI:** 10.1002/nop2.99

**Published:** 2017-09-18

**Authors:** Kristina Wikjord, Vegard Dahl, Signe Søvik

**Affiliations:** ^1^ Faculty of Medicine Institute of Clinical Medicine University of Oslo Oslo Norway; ^2^ Department of Anaesthesia and Intensive Care Akershus University Hospital Lørenskog Norway

**Keywords:** clinical nutrition, critical care, enteral, flow chart, nursing, nutrition support, parenteral, protocol

## Abstract

**Background:**

Enteral nutrition (EN) is associated with improved outcome in critically ill patients and is more affordable. We compared nutritional care practice in our ICU before and after modification of our nutrition support protocol: Several comprehensive documents were substituted with one flow chart and early EN was encouraged.

**Design:**

Retrospective observational study.

**Methods:**

Nutritional data were collected from admission up to 7 days in 25 patients before and 25 patients after protocol modification.

**Results:**

The percentage of patients receiving EN within 72 hr of admission increased from 64% before to 88% after protocol modification. Cumulative percentage energy from EN during ICU days 1–4 increased from 26–89% of total kcal. Overall amount of nutrition administered enterally increased, with a corresponding marked decline in use of parenteral nutrition. Pre‐modification, >80% of patients received >65% of their calculated nutrition requirements by ICU Day 4; post‐modification this goal was achieved by Day 7.

**What does this paper contribute to the wider clinical community?**

- Nutrition support protocols for the ICU should be regularly updated.
- A nurse‐driven nutrition support protocol with easy bedside accessibility and a flow chart design can improve nutritional care for patients in the ICU
- Focus on and knowledge of the benefits of enteral feeding in the ICU team is crucial to increase the early use of enteral nutrition.

## 1 INTRODUCTION

Appropriate nutritional support for critically ill patients is considered a marker of quality in Intensive Care Unit (ICU) care and is associated with improved patient outcome (Heyland, Dhaliwal, Drover, Gramlich, & Dodek, 2003). Despite this knowledge, it has been reported that more than 40% of ICU patients might be malnourished, and the experience from ICUs internationally is that a substantial amount of patients receive suboptimal nutrition (De Jonghe et al., 2001; Kim et al., 2012; McClave et al., 1999).

Critical illness, including traumatic injury, sepsis, burns and major surgery, mobilizes most metabolic pathways and induces a hypercatabolic state. The increased catabolism, diminished oral intake and increased energy expenditure increases the risk of malnutrition in the critically ill patient (Preiser, Ichai, Orban, & Groeneveld, 2014). Also, malnutrition is a common finding in ICU patients already on admission (Hejazi, Mazloom, Zand, Rezaianzadeh, & Amini, 2016).

Current evidence‐based guidelines recommend early (within 48 hr) enteral nutrition (EN) in critically ill patients that are haemodynamically stable and without contraindications to EN (Heyland et al., 2003; Kreymann et al., 2006; Martindale et al., 2009; Seres, 2016). Although the evidence for this recommendation is somewhat conflicting, in balance there seems to be a clinically important reduction in infection and an almost statistically significant reduction in mortality in benefit of early versus delayed EN (Seres, 2016). EN is preferred over parenteral nutrition (PN) because of an associated lower incidence of infection (Heyland et al., 2003; Seres, 2016). The mechanism for this is thought to be through maintenance of gut integrity, gut immune function and modulation of the inflammatory response (Alverdy, Laughlin, & Wu, 2003; McClave & Heyland, 2009). EN is also more cost‐effective than PN.

Protocols for nutritional support have been employed in other ICUs and have been shown to significantly improve nutritional care practice (Dobson & Scott, 2007; Kiss, Byham‐Gray, Denmark, Loetscher, & Brody, 2012; Woien & Bjork, 2006; Wooley & Pomerantz, 2005). Our ICU had used a protocol for nutritional support since 2007. However, as knowledge supporting the benefits of starting EN early and delaying the start of PN in critically ill patients increased, it became clear that the protocol needed modification. Also, feedback from ICU nurses was that the previous protocol was too complicated, unclear and poorly accessible as it consisted of several documents in the institutional electronic procedure database. This likely also contributed to non‐compliance among physicians prescribing nutrition, especially out‐of‐hours when the regular ICU physicians were not present. A modified nutritional care protocol was, therefore, implemented in November 2014. Besides being a simpler and more easily applicable version of previous protocols, the new protocol also‐to a great extent‐limited the use of PN.

The objective of this study was to compare the nutritional care practice before and after the implementation of a modified protocol for nutritional support in the ICU. Our hypothesis was that the proportion of patients receiving at least 65% of calculated nutrition requirements within the first 7 days of ICU admission (Heyland et al., 2003; Kreymann et al., 2006; Martindale et al., 2009; Seres, 2016) would increase and that a larger proportion of the nutrition would be administered through the enteral route. Protocol compliance would be explored by evaluating the proportion of calculated nutrition requirements that was actually prescribed and the proportion of the prescribed energy that was actually administered—although obviously several patient factors not evaluated in our study would result in deviations in these two measures.

## 2 METHODS

### 2.1 Study design and setting

This retrospective observational study was conducted in a 10‐bed medical/surgical ICU in a general emergency university hospital (708 inpatient beds) serving a population of 493,000. The ICU admits approximately 365 patients per year, >90% of which receive mechanical ventilation. Length of stay in 2015 was median 4.0 days; mean 7.6 days. Adult patients admitted to the ICU for ≥72 hr were selected from the patient register in chronological order. While both the previous and the modified protocols calculated energy recommendations from patient body weight (BW), the new nutrition protocol recommended body mass index (BMI)‐stratified energy calculation formulas. Patients from either study period without documented height were therefore ineligible for inclusion and were replaced by the next patient in the patient register.

The pre‐implementation cohort consisted of 25 patients admitted during March–April 2014, the new protocol was implemented in November 2014 and the postimplementation cohort consisted of 25 patients admitted during January–February 2015. The University Hospital Data Protection Authority, in this matter representing the Regional Committee for Medical and Health Research Ethics and the National Data Protection Authority, considered the study exempt from patient consent requirements and granted permission to extract data from patient records.

### 2.2 Development and implementation of a new nutritional protocol

The previous nutritional support protocol had been in use in the ICU since 2007, with updated recommendations for energy prescriptions in 2013. It consisted of several interlinked electronic text documents describing the procedure for nutritional treatment in the ICU, including indications and contraindications for EN and PN, evaluation of nutritional status, calculation of energy requirements, evaluation of degree of gastrointestinal dysfunction, a flow chart for commencement of EN, management of various gastrointestinal symptoms, monitoring of nutritional treatment, use of prokinetics/laxatives/probiotics, nutritional management of special patient groups (severe sepsis, respiratory failure, GI surgery, renal failure, severe pancreatitis, hepatic failure, burns) and types of EN formulas.

**Figure 1:**
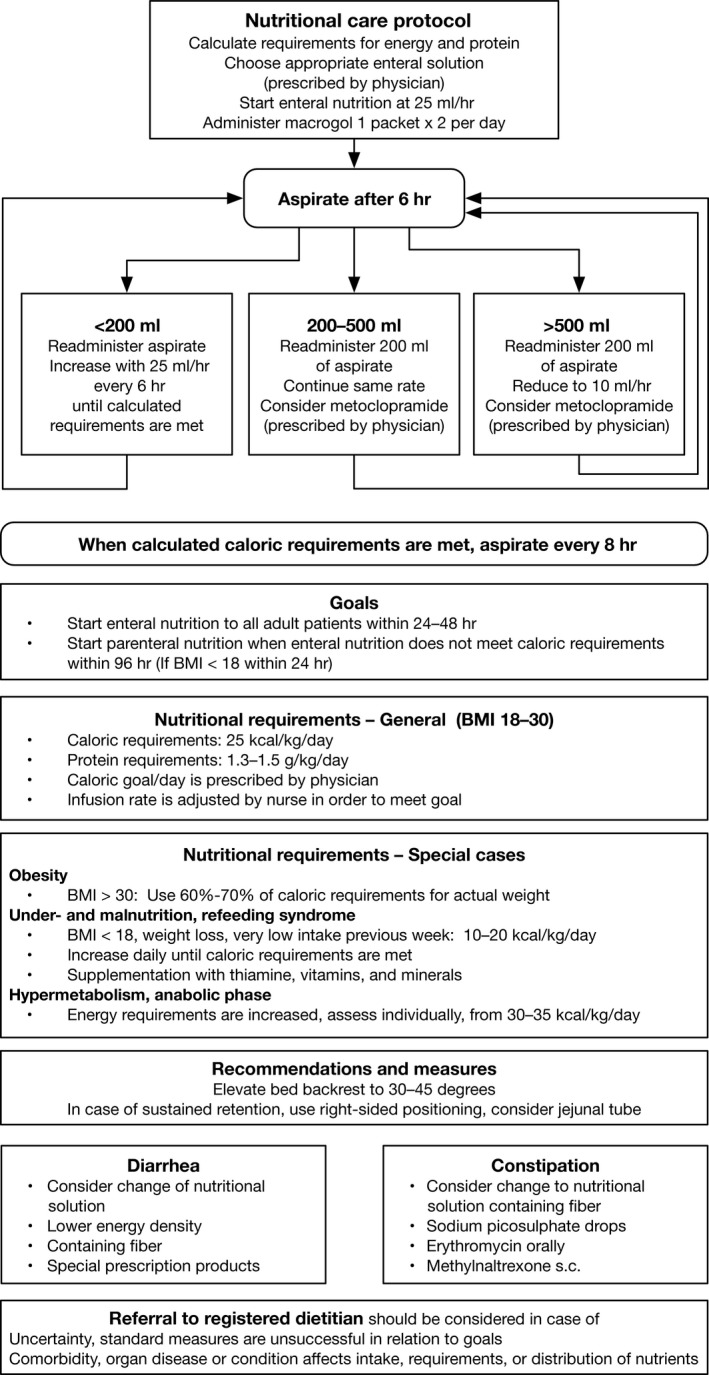
New protocol for nutritional support in the ICU

A standing task group consisting of several ICU nurses and one ICU physician is dedicated to patient nutrition in our department. Unfortunately, our ICU does not have a registered dietitian (RD) service. The task group is available for feedback from ICU staff on the department's nutritional care, discuss and contribute to developing protocols, and is responsible for the continuing education of ICU staff on patient nutrition. In cooperation with the nutrition task group, a modified, flow chart‐based nutrition protocol (Fig. 1) was developed in 2014 by one of the consultant ICU physicians, based on several relevant guidelines and review articles (Casaer et al., 2011; Dhaliwal, Cahill, Lemieux, & Heyland, 2014; Heyland et al., 2003; Kreymann et al., 2006; Martindale et al., 2009; Parrish & McClave, 2008; Seres, 2014a, 2014b; Singer et al., 2009).

On several aspects, the new nutritional protocol differed from the previous protocol. Although the previous protocol recommended supplementation with PN after 2–3 days if <80% of energy requirements were met, the new protocol delayed supplementation with PN to 96 hr after admission. The previous protocol recommended administering only intravenous fluids containing glucose the first 24 hr of ICU admission; in contrast, the new protocol recommended starting early EN (within 24–48 hr). The new protocol also recommended BMI‐stratified calculation of energy requirements, with lower goals of kcal/kg for obese and underweight patients (Heyland et al., 2003; Kreymann et al., 2006; Martindale et al., 2009; Seres, 2016).

The new protocol described the process of enteral feeding and included recommendations on timing of feeding, patient positioning, management of obstipation/diarrhoea and GI retention and supplementation with PN. It consisted of a single‐page flow chart and one page with specifications. The process was nurse‐driven, the default being that every ICU patient should be evaluated for EN within 24 hr. If no EN was prescribed and no contraindications were obvious, the ICU nurse would contact the physician to enquire about this. According to protocol, nurses prescribed and administered multivitamins and makrogol and adjusted the rate of feeding to tolerance and to obtain the energy goals prescribed by the physician. Pauses in EN, for example, due to imaging procedures, were compensated for by increasing feeding rates afterwards.

Preceding the implementation, the ICU nurses were educated in the use of the protocol and on current nutritional care recommendations. The protocol was posted on the wall of all ICU patient rooms as well as in the storage room for EN formulas. All regular ICU physicians were thoroughly informed on the new protocol. However, there was no formalized education of the roster of anaesthesiologists responsible for the ICU at late evening and night.

### 2.3 Definition of variables

Investigated variables were: Amount of kcal prescribed and administered each of the first 7 days of admittance, or until discharge from the ICU for patient stays <7 days. Amount of kcal administered through the parenteral and the enteral route. Patient gender, age, height, BW on admission, BMI and length of ICU stay were also noted. Data were collected retrospectively from the ICU patient register and electronic medical records.

Prescribed energy was the amount of kcal set as nutritional goal for the upcoming 24 hr (morning to next morning), entered each day in the electronic patient chart by the attending physician. For ICU patients, energy prescriptions should be based on patient BW but must also take into account their clinical situation. We suspected, however, that prescribing practices might differ among the physicians, also due to lack of knowledge or non‐compliance with the protocol. Therefore, we wanted to assess the actual prescribed energy as proportion of the calculated nutrition requirements, for the two periods. Energy requirements in this study were calculated according to the BMI‐stratified, BW‐based equations in the new nutrition protocol (Fig. 1). Default value was 25 kcal kg^−1^ day^−1^. For obese patients (BMI>30), energy requirements were reduced to 65%, that is, 16.5 kcal kg^−1^ day^−1^. For underweight patients (BMI<18) nutrition requirements for evaluations in this study was set to 15 kcal kg^−1^ day^−1^, throughout ICU Days 1–7 (the protocol recommended 10–20 kcal kg^−1^ day^−1^). Energy prescribed and energy administered were compared with the calculated goals, realizing that deviations could have very justifiable clinical reasons. The amount of kcal administered as EN and as PN was reported also as percentage of total administered energy.

Primary end‐points were commencement of EN within 72 hr, cumulative energy relative to individual requirements administered during the first week after ICU admission and percentage of cumulative energy administered via the enteral route. For each day after admission we measured the fraction of patients receiving at least 65% of calculated daily energy requirements.

### 2.4 Statistical analysis

Pre‐ and postimplementation values of variables measured repeatedly in subjects were compared using a mixed model linear regression with subject as random factor (JMP 11.2.1 by SAS Institute Inc), after exclusion of any extreme outliers (<2% of datapoints). Two‐group comparisons of non‐repeated variables were performed with Wilcoxon test or Chi Square test as appropriate. Level of significance was set at *p *=* *0.05. Distributions of variables were described by medians (25th–75th percentiles) if not otherwise specified.

## 3 RESULTS

### 3.1 Study population

Patient characteristics are presented in Table 1. The two groups were comparable on all reported demographic variables. In the inclusion process of the 25 + 25 patients, six patients in the pre‐modification period and three patients in the postmodification period had to be bypassed due to undocumented height.

**Table 1:** Patient and clinical characteristics

	Pre‐implementation (*N *=* *25)	Post‐implementation (*N *=* *25)	*p* value^a^
Admission reason			
Medical	18 (72)	17 (68)	
Surgical	7 (28)	8 (32)	.758
Sex			
Male	18 (72)	13 (52)	
Female	7 (28)	12 (48)	.145
Age (years)	63.5 (34.3–80.2)	65.9 (32.8–83.7)	.808
Length of ICU stay (days)	10 (3–65)	10 (3–24)	.915
Body mass index (kg/m^2^)	27.5 (15.4–53.8)	30.9 (16.8–54.7)	.118
Calculated requirement (kcal)	1825 (555–2440)	1750 (720–2275)	.351

Values are No. (%) or Median (min–max).

a Wilcoxon or Chi Square test, as appropriate.

### 3.2 Nutritional care practice

**Figure 2:**
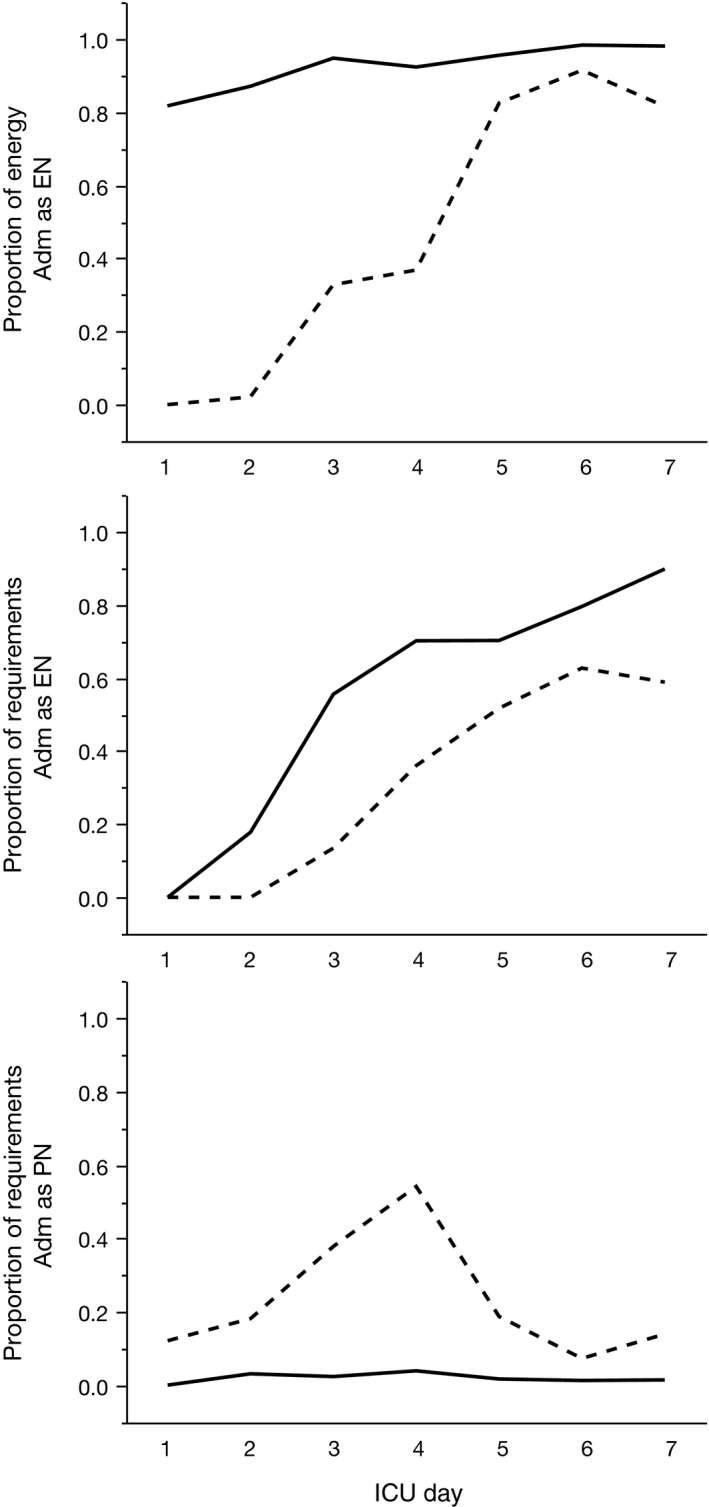
Effects of introducing a modified, flow chart‐based, nurse‐driven nutrition protocol emphasizing enteral nutrition. Lines represent group median values from 25 ICU patients *before* (dashed lines) and 25 ICU patients *after* (continuous lines) protocol implementation. Upper panel: Proportion of total energy (Kcal) administered enterally. Middle panel: Proportion of individual calculated nutrition requirements administered as enteral nutrition. Lower panel: Proportion of individual calculated nutrition requirements administered as parenteral nutrition

Cumulative energy administration results are presented in Table 2 and Fig. 2.

**Table 2:** Cumulative energy administration

	Pre‐implementation (*N* = 25)	Post‐implementation (*N* = 25)	*p* value^a^
Commenced EN			
Within 24 hr	6 (24)	12 (48)	.075
Within 48 hr	12 (48)	15 (60)	.395
Within 72 hr	16 (64)	22 (88)	.043
Cumulative total energy			
ICU day 1–4	4547 (3554–5499)	2986 (2654–5370)	.091
% of requirements	38% (27–52)	39% (22–50)	.458
Cumulative EN			
ICU day 1–4	880 (112–3761)	2673 (1531–4532)	.014
% of total energy	26% (4–79)	89% (72–100)	.0002
Cumulative PN			
ICU day 1–4	2362 (937–4459)	211 (0–1216)	<.0001
% of total energy	74% (21–96)	15% (0–29)	.0002
Cumulative total energy			
ICU day 1–7	9172 (7996–10756)	8128 (5430–10492)	.1980
% of requirements	71% (63–82)	71% (51–100)	.817

Energy measured as kcal. Calculated individual requirements are 25 kcal kg^−1^ day^−1^ if BMI 18–30; 15 kcal kg^−1^ day^−1^ if BMI<18; 16.5 kcal kg^−1^ day^−1^ if BMI>30.

Values are No. (%) or Median (25th–75th percentiles).

a Wilcoxon test or Chi Square test, as appropriate.

#### 3.2.1 Prescribed energy

The proportion of calculated nutrition requirements that was prescribed tended to increase after protocol modification, from 0.9 (0.81–1.03) to 1.0 (0.85–1.23) (mixed model linear regression; *p *=* *0.06). Median prescribed kcal was 1600 (1500–1800) pre‐implementation and 1600 (1400–2000) postimplementation (mixed model linear regression; *p *=* *0.57). For underweight patients (BMI <18), there was no correlation between BW and physician‐prescribed energy, while for normal‐weight and overweight patients, physician‐prescribed energy as expected correlated positively with BW. Still, in both study periods, there was a negative relation between the calculated requirements and the proportion of these energy requirements that was actually prescribed (mixed model linear regression; *p *<* *0.001; *R* ^2^ = 0.92), that is, lower‐weight patients were relatively overfed and heavier patients with normal BMI relatively underfed. As a result, for two hypothetical patients with calculated nutrition requirements of 2500 kcal/d and 1000 kcal/d, the expected energy prescriptions would be approximately 50% and 150% of energy requirements, respectively. The band of variation of prescribed energy was wide, approximately 1000 kcal/d for patients with identical BW.

#### 3.2.2 Total energy administered

**Figure 3:**
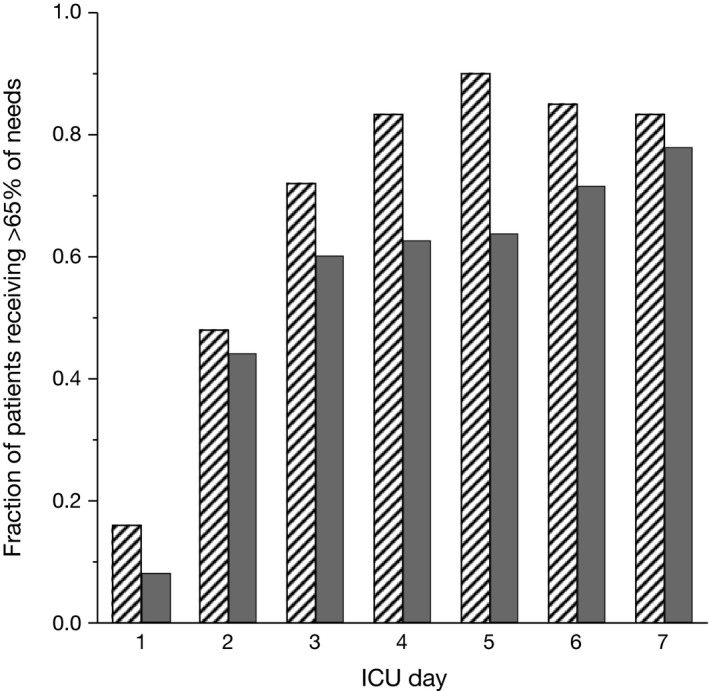
Proportion of ICU patients receiving at least 65% of their calculated nutrition requirements, enterally and/or parentally, during their first ICU week. Data from 25 patients before (Hatched columns) and 25 patients after (Black columns) introduction of a modified nutrition protocol emphasizing early enteral feeding and delayed introduction of parenteral nutrition

In both study periods, administered energy increased day by day after ICU admission (mixed model linear regression; *p *<* *0.0001), with large inter‐individual variations (Fig. 2). The cumulative amount of kcal administered was unchanged after protocol modification, during ICU days 1–4 as well as during the entire 7‐day period after admission (Table 2). Also measured as proportion of calculated nutrition requirements, cumulative energy administered was unchanged after protocol modification (Table 2). With the previous protocol, approximately 80% of patients received >65% of their calculated nutrition requirements by ICU Day 4. This goal was achieved by Day 7, postmodification (Fig. 3).

**Figure 4:**
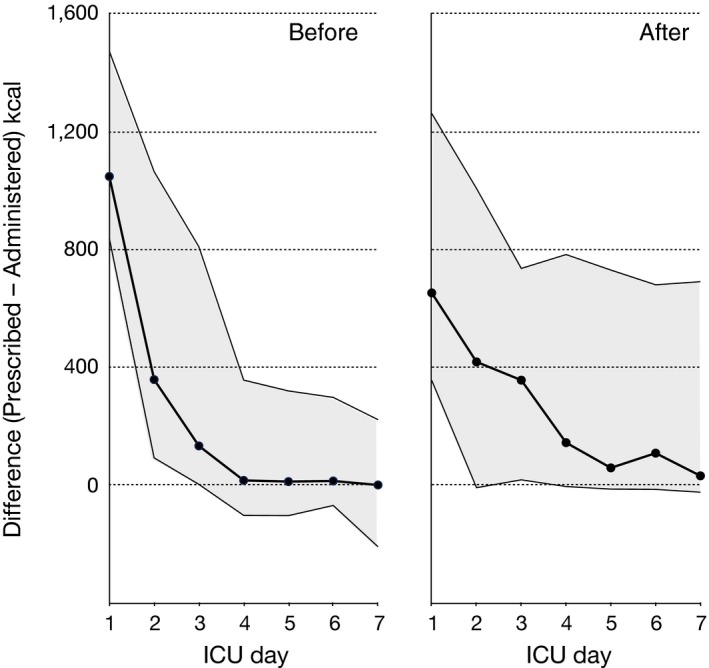
Difference between prescribed energy and administered energy (kcal) in 25 ICU patients before and 25 patients after introduction of a modified nutrition protocol emphasizing early enteral nutrition (EN) and delayed introduction of parenteral nutrition (PN). Data are group medians, grey‐shaded areas indicate 25th–75th percentile ranges. The modified protocol resulted in larger deviations, indicating that nutritional goals were harder to reach with EN than PN

The proportion of the prescribed energy actually administered to the patient increased day by day after ICU admission in both study periods (mixed model linear regression; *p *<* *0.001), but overall was approximately 10% less after protocol modification (*p *=* *0.05). After introduction of the modified protocol emphasizing use of EN, the difference between nutritional goals and actual received nutrition was larger for more patients (Fig. 4). This difference was of similar magnitude throughout all the first ICU week.

#### 3.2.3 Administration route

The proportion of patients that had commenced enteral feeds within 72 hr of ICU admission increased from 64% before to 88% after protocol modification (Table 2). A similar trend was seen already after 24 hr (Table 2). Correspondingly, the proportion of administered energy that was administered enterally during the first days after ICU admission was markedly higher with the modified protocol (Fig. 2 Upper panel). Also, the proportion of calculated nutrition requirements that was administered through the enteral route increased more rapidly after ICU admission and rose to higher levels (Fig. 2 Middle panel). Concurrently, there was a marked decline in the use of PN (Fig. 2 Lower panel). The cumulative percentage energy given as EN during ICU days 1–4 increased from 26% pre‐ – 89% postimplementation (Table 2).

## 4 DISCUSSION

This study demonstrated a marked change in nutritional care practice in an ICU 4 months after modification of a nutritional support protocol, from several comprehensive, electronically stored documents to a simple flow chart, posted bedside and with nurse‐driven escalation of feeding rates. The recommendations in the modified protocol concerning early enteral nutrition were clearly adhered to, while physician‐prescribed energy in relation to recommended calculated requirements was variable.

### 4.1 Prescription of nutrition

Our results showed no significant change in physician‐prescribed kcal after implementation of the new protocol. In both study periods, there was a consistent negative relation between the nutrition requirements calculated from BW and the proportion of these requirements that was actually prescribed. Interestingly, this effect was consistent throughout ICU day 1–7, indicating that ICU physicians and nurses on new shifts tended to accept and extend previous energy prescriptions, even when these conflicted with the nutrition protocol. Our data show that physicians systematically tended to increase energy administration in patients with low BMI and to restrict energy administration in obese patients. Still, prescriptions varied by as much as 1000 kcal/day among patients with similar BW. Thus, our findings indicate that prescribing practice might not be adequately individualized and that physicians might still have been using a “one‐size‐fits‐all” prescription instead of calculating individual energy requirements. An important reason underlying this finding probably was the lack of an education plan regarding the new nutritional support protocol for physicians that served the ICU only sporadically or on evenings and nights. Conceivably, our electronic patient curve could have been programmed to display each ICU patient's calculated energy requirements according to the new protocol—this probably would have improved protocol adherence.

Several barriers to guideline implementation have been described (Fischer, Lange, Klose, Greiner, & Kraemer, 2016) and adherence to the nutritional support protocol in our ICU would likely improve further if such factors were regularly attended to. Systematized and continuous interprofessional teamwork including nurses, registered dietitians (RDs) and ICU physicians is crucial to successfully implement and uphold good nutrition practice. We did not include data on clinical conditions, which must have affected prescription behaviour. Importantly, prescription based solely on weight‐ and BMI‐derived calculated requirements does not take into account individual aspects regarding disease progress and does not sufficiently support, for example, increased needs in the anabolic phase (Kreymann et al., 2006).

### 4.2 Administration of nutrition

In the postimplementation group, there was a trend towards a small decrease in cumulative energy administered over the first 4 days after ICU admission (*p *=* *0.09) and it took longer for patients to reach >65% of their energy requirements (Fig. 3). This probably resulted from the more gradual increase in administration of enteral feeds that is necessary compared with what is often the case with PN. In the acute phase of critical illness it may be difficult to reach the calculated caloric requirements with EN alone due to impaired GI mobility. This likely underlay the findings shown in Fig. 4, where the postmodification group showed larger discrepancies between physician‐prescribed energy goals and actual administered nutrition. However, in several recent studies, hypocaloric feeding in critically ill patients was not shown to be harmful (Arabi et al., 2015; Charles et al., 2014; Choi, Park, & Park, 2015; Ibrahim et al., 2002; Rice et al., 2011, 2012). A lower calorie provision in the initial phase of critical illness is therefore not necessarily detrimental and may be beneficial (Al‐Dorzi, Albarrak, Ferwana, Murad, & Arabi, 2016); an adequate goal may be to deliver 50%–65% of calculated nutritional requirements as EN within the first ICU week (Martindale et al., 2009). Moreover, the amount of delivered protein, not energy, may be key for critically ill patients (Compher, Chittams, Sammarco, Nicolo, & Heyland, 2017).

### 4.3 Route of nutrition

The new protocol resulted in a marked increase in the overall amount of nutrition administered through the enteral route, as well as an increase in patients receiving early EN (Fig. 2). This change in feeding practice is considered favourable and in accordance with current recommendations (Heyland et al., 2003; Kreymann et al., 2006; Martindale et al., 2009; Seres, 2016). These recommendations are based on several prospective randomized, controlled trials and meta‐analyses involving various ICU patient populations, and a consistent reduction in infectious morbidity is found (Braunschweig, Levy, Sheean, & Wang, 2001; Elke et al., 2016; Everitt, 1998; Gramlich et al., 2004; Heyland et al., 2003; Kudsk et al., 1992; Martindale et al., 2009; Moore et al., 1992; Peter, Moran, & Phillips‐Hughes, 2005; Simpson & Doig, 2005). In many studies further benefits are seen: Reduction in hospital length of stay (Heyland et al., 2003), cost of nutrition therapy (Heyland et al., 2003) and return of cognitive function in head injury patients (Taylor, Fettes, Jewkes, & Nelson, 1999). This was not investigated in our small study. From a global perspective, increased use of EN in ICU patients is attractive also due to lower cost and no need for long‐term central venous catheters, which predispose patients for infection and vascular complications.

Several other studies (Doig et al., 2008; Heyland et al., 2010; Kiss et al., 2012; Mackenzie, Zygun, Whitmore, Doig, & Hameed, 2005; Martin, Doig, Heyland, Morrison, & Sibbald, 2004; Woien & Bjork, 2006) have investigated the effect of implementation of nutrition protocols in the ICU. In most of these studies, there was no existing protocol for nutritional care in the ICU before implementation, which makes them not completely comparable to our study. A systematic review (Martin et al., 2004) including three prospective cohort studies and one clustered RCT showed increased efficacy of enteral feeding delivery with nutritional support protocols. In all studies reviewed, there was increased energy delivery, increase in the use of enteral versus parenteral nutrition, mixed results in time to initiation of feeding and no difference in patient outcome.

### 4.4 Strengths and limitations

Our study was limited by its small number of participants; this could increase the risk of bias. Patients were unselected with regard to gastrointestinal morbidity that could impede nutritional efforts, and inter‐individual variation was indeed large. Even so, the observed changes in clinical practice were marked. The retrospective design and the spacing in time from baseline measures to protocol implementation and from implementation to evaluation of protocol adherence would have minimized the Hawthorne effect, that is, that study subjects optimize their practice because they know they are being studied. On the other hand, any documentation errors leading to inaccurate amounts of various nutrients being registered in the electronic patient curve would go undetected by our retrospective approach. Our material was too small to study whether the implementation of the nutrition protocol with the following changes in practice made any impact on patient mortality or morbidity, that is, infection risk. Scores for severity of disease (i.e. SAPS or APACHE) of the participants was not investigated in this study. Possible differences in these parameters between the groups and the relation between calorie prescription or administration and severity of disease could therefore not be studied.

## 5 CONCLUSION

Implementation of a flow chart‐based, nurse‐driven nutritional support protocol in the ICU resulted in more appropriate nutritional support according to current guidelines, with a significant increase in the early use of enteral feeding and reduced use of PN. A delay in reaching goals for total administered energy was observed during the first week after ICU admission. Further studies are needed to clarify energy and protein needs and the optimal timing of nutrient administration in the critically ill patient. Interprofessional teamwork is key for successful implementation of nutrition support in the ICU.

## 6 RELEVANCE TO CLINICAL PRACTICE

Implementation of a simple, flowchart‐based protocol can improve the nutritional care of patients in intensive care units. Our study indicates that accessibility and user‐friendliness of the nutrition care protocol and focus and knowledge on the benefits of enteral feeding among ICU staff probably were key factors in providing optimal nutrition to ICU patients.

## CONFLICTS OF INTEREST

The authors hereby declare that they have no financial or proprietary interest in the subject matter or materials discussed in the manuscript, including (but not limited to) employment, consultancies, stock ownership, honoraria and paid expert testimony. Neither do the authors have non‐financial conflicts of interest to declare.

## AUTHOR CONTRIBUTIONS

KW, VD and SS planned and designed the study. KW collected the data. KW and SS carried out the statistical analyses. KW drafted the manuscript and created the tables, SS created the figures. All authors evaluated and discussed the ongoing analyses, critically revised the manuscript and approved the final version.

All the Authors have agreed on the final version and meet at least one of the following criteria [recommended by the ICMJE (http://www.icmje.org/recommendations/)]:

- substantial contribution to conception and design, acquisition of data or analysis and interpretation of data;
- drafting the article or revising it critically for important intellectual content.
